# Epigenetics in neonatal necrotizing enterocolitis: current understanding and the potential involvement of m6A modification

**DOI:** 10.3389/fped.2025.1617042

**Published:** 2025-09-02

**Authors:** Yixian Chen, Yujun Chen

**Affiliations:** ^1^ Neonatology, Liuzhou Hospital of Guangzhou Women and Children's Medical Center, Guangxi, China; ^2^ Department of Pediatrics, The Second Affiliated Hospital of Guangxi Medical University, Guangxi, China

**Keywords:** necrotizing enterocolitis, epigenetics, N6-methyladenosine, neonates, DNA methylation

## Abstract

Necrotizing enterocolitis (NEC) exhibits high incidence, surgical intervention rates, and mortality among preterm infants, profoundly impacting survivor's long-term quality of life. Consequently, the etiology and pathogenesis of this disease remain incompletely elucidated. Emerging evidence underscores the intricate connection between epigenetics and NEC. DNA methylation, histone modifications, and non-coding RNAs regulate disease development through targeted modification of transcriptional regulation and translational control in NEC-associated genes, thereby driving pathological progression. Notably, N6-methyladenosine (m6A) modification, the most prevalent form of RNA epigenetic regulation, exerts critical functions in intestinal inflammation, microbial homeostasis, and injury repair, suggesting its potential involvement in NEC development. In this review, we will summarize the current mechanistic understanding of NEC, emphasizing its interplay with epigenetics (DNA methylation, histone modifications, and non-coding RNAs). we also explore the emerging role of m6A RNA modification in gut pathophysiology, proposing its potential role in NEC.

## 1 Introduction

Necrotizing enterocolitis (NEC), a life-threatening gastrointestinal emergency in neonates, demonstrates an incidence rate of 2%–13% among preterm infants, with the global average reaching 7% in very low birth weight (VLBW) infants (1). The overall mortality rate stands at 29.3% in preterm neonates, escalating to 32.5% in VLBW infants with NEC (2). Approximately 41.7% of NEC cases require surgical intervention, accompanied by a striking 34.9% mortality rate in surgically managed patients (3). Survivors frequently develop long-term sequelae, including growth retardation, neurodevelopmental delays, short bowel syndrome, intestinal strictures/adhesions, and cholestasis (4). Therefore, elucidating the pathogenesis of NEC and identifying its risk factors are critical for developing effective prevention and treatment strategies. However, the precise etiology and pathogenesis of NEC remain incompletely understood, though current evidence confirms its multifactorial nature involving intestinal immaturity, formula feeding, microbial dysbiosis, and infections (5).

**Figure 1:**
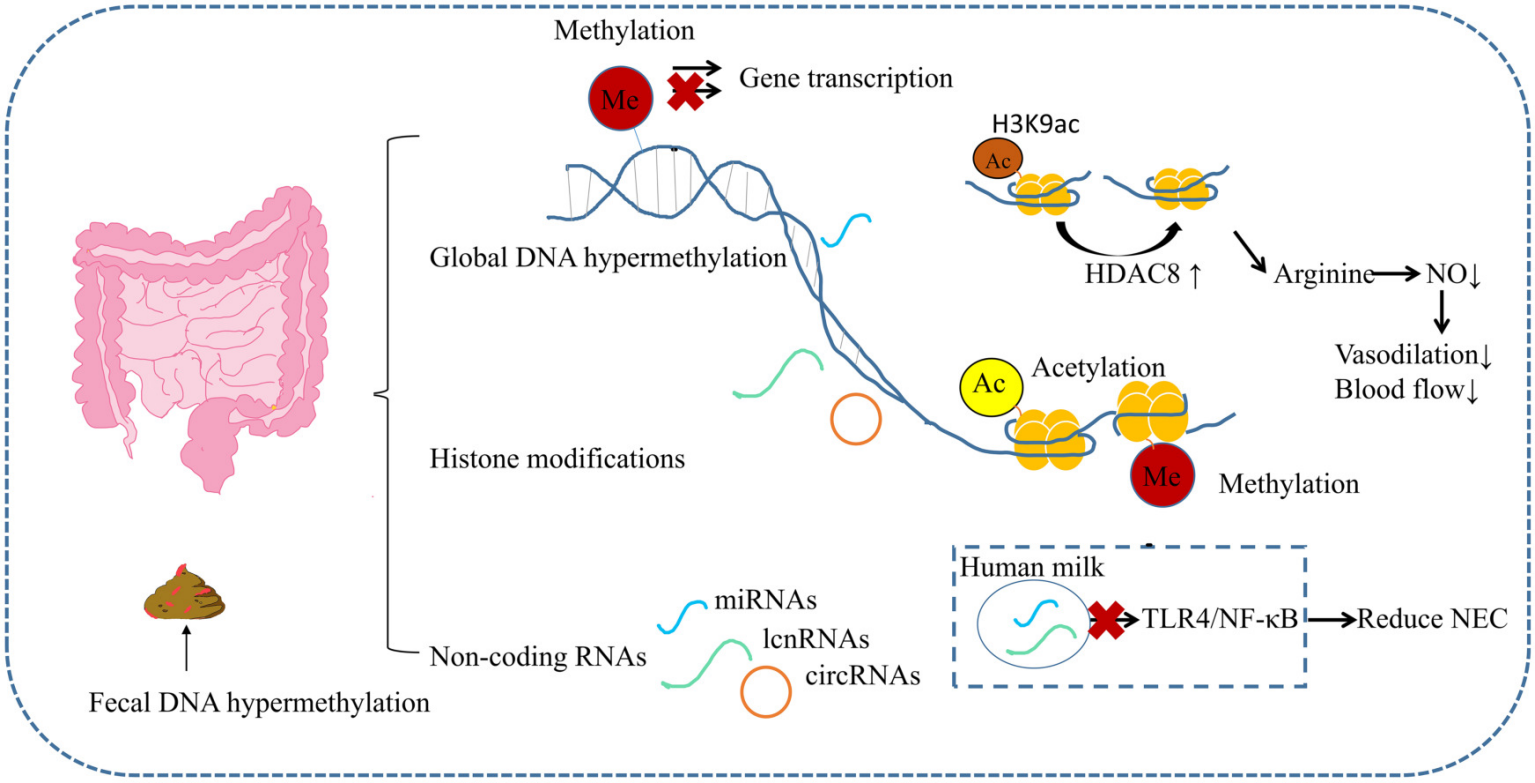
Epigenetics in NEC. Gut and feces from infants with surgical NEC exhibit widespread DNA hypermethylation. Dysregulation of histone modifiers is evident in NEC. For instance, HDAC8 upregulation deacetylates H3K9ac at promoters of arginine metabolism genes, repressing their expression and reducing arginine-dependent NO synthesis. Differentially expressed ncRNAs function as epigenetic regulators by recruiting chromatin complexes or binding mRNAs. Milk-derived ncRNAs inhibit TLR4/NF-κB signaling, attenuating NEC severity.

Epigenetics bridges environmental exposures with genomic regulation, wherein external factors (nutrition, infection, pharmacological agents, and physiological stress) induce nucleotide base modifications or protein alterations without altering DNA sequences, thereby regulating gene expression through heritable and reversible mechanisms (6, 7). Studies have found that NEC involves multiple epigenetic mechanisms. DNA methylation, histone modification, and non-coding RNAs are involved in the occurrence and development of NEC (Figure 1), affecting the disease phenotype (8). This review delineates the current research landscape of epigenetic mechanisms in NEC, discuss the potential mechanisms of m6A modification in NEC, providing a basis for clarifying the etiology and pathogenesis of NEC, ultimately providing mechanistic insights to inform targeted therapeutic development.

## 2 Overview of the pathogenesis of NEC

TLR4 (Toll-like receptor 4) serves as the primary pattern recognition receptor for bacterial endotoxin (lipopolysaccharide, LPS) and functions as a central mediator in the pathogenesis of NEC (9). LPS from gram-negative bacteria in the intestinal lumen subsequently binds to TLR4 expressed on intestinal epithelial cells (IECs), initiating a cascade of downstream signaling events, ultimately driving the excessive production of pro-inflammatory cytokines such as tumor necrosis factor-α (TNF-α) and interleukin-1β (IL-1β) (10). The ensuing cytokine storm exacerbates disruption of the mucosal barrier and causes local tissue damage. Subsequent translocation of gut-derived bacteria and their pathogenic metabolites across the compromised intestinal epithelium into systemic circulation leads to TLR4 activation on vascular endothelial cells. This receptor engagement suppresses endothelial nitric oxide synthase (eNOS) expression, thereby triggering mesenteric vasoconstriction through impaired NO-mediated vasodilation (10). These pathophysiological cascades culminate in intestinal hypoperfusion, transmural necrosis, and ultimately bowel perforation (9).

During the initial phase of NEC, clinical presentations frequently include gastrointestinal dysfunction characterized by emesis, abdominal distension, and enteral feeding intolerance, representing manifestations of impaired gastrointestinal motility (11). Gastrointestinal motility is mainly regulated by enteric nervous system (ENS) composed of neurons and enteric glial cells (EGCs). There are abundant EGCs surrounding enteric neurons in the intestinal mucosa. Structural or functional impairment of EGCs disrupts ENS, thereby contributing to dysregulated intestinal motility (12). Kovler et al. (12) conducted a multispecies histopathological analysis of ileal specimens from NEC mice, porcine models, and human neonates, demonstrating a marked depletion of EGCs across all study cohorts. Mice with EGCs deficiency exhibited significantly higher susceptibility to severe NEC phenotypes compared to wild-type controls. Mechanistically, conditional knockout of TLR4 specifically in EGCs attenuated the EGCs depletion observed in experimental NEC, suggesting TLR4 signaling directly mediates EGCs loss during disease progression. EGCs exert protective effects against NEC through brain-derived neurotrophic factor (BDNF) mediated suppression of TLR4 activation, thereby mitigating NEC initiation and progression (12).

TLR9, a structural homolog of TLR4, is significantly downregulated in intestinal tissues from NEC (13). Mechanistic studies reveal that TLR9 activation enhances expression of interleukin-1 receptor-associated kinase-M (IRAK-M), a negative feedback regulator of TLR4 signaling, attenuates TLR4-driven inflammatory cascades, ultimately ameliorating NEC severity (14). Thus, the dynamic interplay between TLR4 and TLR9 signaling pathways in the neonatal gut contributes critically to the pathogenesis and progression of NEC (10). Furthermore, accumulating evidence has established a causal link between gut microbiota dysbiosis and NEC. A recent meta-analysis investigating gut microbial dysbiosis in preterm infants prior to NEC onset revealed a significant enrichment of *Proteobacteria* coupled with reduced relative abundances of *Firmicutes* and *Bacteroidetes* during the pre-NEC phase (15).

## 3 DNA methylation and NEC

### 3.1 DNA methylation patterns in clinical NEC

DNA methylation is an enzymatic process catalysed by DNA methyltransferases (DNMTs), involving the covalent addition of a methyl group (–CH₃) from S-adenosylmethionine (SAM) to the C5 position of cytosine residues within CpG dinucleotide sequences (6). A meta-analysis including 3,648 neonatal samples from 17 distinct cohorts revealed that methylation at 8,899 CpG sites within 4,966 genes in umbilical cord blood was significantly associated with neonatal gestational age. Significant correlations have been observed between DNA methylation patterns in umbilical cord blood, brain, and lung tissues of fetuses at comparable gestational ages (16). The study by Good et al. (17) demonstrated that ileal and colonic tissues from infants with surgical NEC exhibited widespread DNA hypermethylation. A total of 7,087 differentially methylated sites were associated with genes showing significant transcriptional differences. Among promoter regions of downregulated genes in NEC, 92% of differentially methylated sites displayed hypermethylation, while this proportion decreased to 66% in upregulated genes (18). Tian et al. (18) performed multi-omics analyses integrating methylation profiling, RNA transcriptome, and single-cell sequencing data from NEC intestinal tissues. Their investigation revealed differentially expressed genes exhibiting an inverse correlation between promoter region methylation levels and transcriptional activity. These genes demonstrated predominant enrichment within the intestinal epithelial compartment, and were functionally associated with the modulation of T cell differentiation processes and suppression of intestinal inflammatory responses (18).

Moreover, studies have revealed distinct DNA methylation patterns in fecal samples from infants with NEC, with a marked correlation observed between the methylation profiles of fecal specimens and colonic tissues (19, 20). Klerk et al. (19) conducted a comparative analysis of fecal DNA specimens from 24 NEC and 45 non-NEC preterm infants, systematically comparing methylation levels of multiple genes across pre-onset, acute, and post-onset phases. Their findings demonstrated significantly elevated methylation at the TLR4 CpG2 site in infants with NEC compared to non-NEC controls. Within the NEC cohort, the methylation of the vascular endothelial growth factor A (VEGFA) CpG2 site was 0.8% for a long time before NEC, increased to 1.8% shortly before NEC, and increased to 2.0% after NEC. An analogous pattern was observed at the DEFA5 CpG1 site, where methylation rates progressed from 75.4% to 81.4% (19). Moreover, investigations have identified that during the pre-NEC phase, fecal DNA from infants with NEC exhibited C-terminal domain small phosphatase-like 2 (CTDSPL2) methylation levels of 51%, demonstrating a statistically significant elevation compared with non-NEC controls (17%) (21). These findings collectively suggest that fecal DNA methylation profiling holds promise as a predictive biomarker for NEC progression. Furthermore, the targeted analysis of specific methylation sites may provide novel therapeutic targets for both preventive interventions and clinical management of NEC.

### 3.2 Mechanistic involvement of DNA methylation in NEC

In mammals, the DNA methylation process is mainly regulated by DNA methyltransferases DNMT1 and DNMT3, which serve distinct functions: DNMT1 primarily maintains existing methylation patterns during DNA replication (maintenance methylation), while DNMT3 establishes new methylation marks (*de novo* methylation) (22). Within the intestinal epithelium, DNMT1 demonstrates spatial specificity, exhibiting predominant localization to the crypt base proliferative zone. Targeted deletion of DNMT1 in intervillus progenitor cells triggers a cascade of pathobiological consequences: genomic hypomethylation, DNA damage premature differentiation and apoptosis of progenitor cells, and ultimately the loss of nascent villi (23). Profound disruptions in intestinal crypt homeostasis caused by apoptosis (24) and abnormal differentiation of stem cells (25), along with necrosis and loss of villi (24), all of which are also observed in NEC. Research has found that the methylation degree of the TLR4 in differentiated IECs is significantly higher than that in undifferentiated IECs, suggesting that the methylation of TLR4 is involved in the differentiation process of IECs, and this process is mainly regulated by DNMT3 (26). DNA methylation mediates downregulation of TLR4 through promoter hypermethylation, leading to attenuated LPS responsiveness in IECs (27). Animal studies have demonstrated that prenatal intrauterine infection induces alterations in DNA methylation patterns within the promoter regions of multiple genes participating in TLR4 signaling pathway in the small intestine of offspring (7). Cortese et al. (28) demonstrated that exposure of immature IECs to probiotic or pathogenic bacteria results in the identification of over 200 differentially modified DNA regions, indicating that early microbial colonization may induce alterations in intestinal epigenetic signatures. Routine postnatal oral antibiotic administration in preterm piglets delays microbial colonization and alters genomic DNA methylation, potentially influencing intestinal health by modulating the expression of genes linked to intestinal immunity, vascular integrity, and metabolism, thereby contributing to NEC prevention (29). CTDSPL2 hypermethylation precedes NEC onset, suppressing its transcription via DNA methylation, thereby impairing cell cycle progression and exacerbating inflammation through NF-κB signaling (21). Therefore, prenatal and postnatal environmental factors may exert their influence on intestinal development and homeostasis through modulation of genomic DNA methylation in the gut, potentially representing one of the key mechanisms underlying the predisposition to NEC in preterm infants (30).

## 4 Histone modifications and NEC

### 4.1 Histone modifications patterns in NEC

The nucleosome, composed of DNA and histones, is the basic structural unit of chromatin. When the N-terminus of the histone tail is modified, the state of chromatin is affected, and thus the expression of genes is changed. Such histone modifications include acetylation, methylation, phosphorylation, and ubiquitination (9). Differential expression of histone methylation regulating genes has been demonstrated in intestinal tissues of infants with NEC, with the most pronounced alteration observed in KDM6B. Both upregulated JMJD3 (encoded by KDM6B) and reduced H3K27 trimethylation (H3K27me3) were more evident in NEC patients and animal models compared to controls (31). In addition, histone deacetylase 8 (HDAC8) is highly expressed in NEC (32).

### 4.2 Mechanistic involvement of histone modifications in NEC

Studies have demonstrated that histone modifications are integral to maintaining intestinal homeostasis, with the associations between histone acetylation/methylation and NEC having been conclusively established (33). In dextran sodium sulfate (DSS) induced murine colitis models, H3K27ac levels are significantly downregulated, whereas histone HDAC inhibitors restore H3K27ac levels and suppress pro-inflammatory cytokine production (33). In NEC, upregulated HDAC8 suppresses transcriptional activity by reducing H3K9ac levels at the promoter regions of key arginine metabolic pathway genes (PRODH/PRODH2), thereby diminishing arginine biosynthesis (32). Arginine serves as the precursor for endogenous NO synthesis, with NO playing a critical role in NEC pathogenesis by modulating intestinal vasodilation and blood flow (34). Selective inhibition of HDAC8 ameliorates arginine metabolism disorders, alleviates intestinal injury in NEC mouse model, and attenuates the production of pro-inflammatory factors IL6, IL-1β, and TNF-α (32). Histone hyperacetylation maintains chromatin in an open state at the TLR4 gene promoter region, facilitating transcription. The use of HDAC inhibitors can enhance TLR4 expression in IECs (35). The administration of GSK-J4, a specific inhibitor of JMJD3, effectively mitigated the overexpression of JMJD3 and reversed the hypoexpression of H3K27me3 in NEC mice. Subsequently, it exerted its anti-inflammatory effects by suppressing inflammatory responses via the NF-κB and JAK2/STAT3 signaling pathways. Moreover, GSK-J4 ameliorated intestinal injury through the inhibition of necroptosis, ultimately contributing to the alleviation of NEC (31). These findings suggest that targeting histone-modifying enzymes involved in histone acetylation and methylation, could be a potential strategy for the prevention and treatment of NEC.

## 5 Non-coding RNAs and NEC

### 5.1 Non-coding RNAs patterns in NEC

Non-coding RNAs function as multifunctional regulators by interacting with DNA, RNA, and protein complexes to modulate gene expression at multiple regulatory levels, spanning transcriptional regulation to post-transcriptional modifications (36, 37). Comparative analyses of plasma and intestinal tissue samples between NEC and non-NEC infants have systematically identified differentially expressed ncRNAs, particularly microRNAs (miRNAs), long non-coding RNAs (lncRNAs), and circular RNAs (circRNAs) (38–40). Han et al. (38) performed whole-transcriptome sequencing of miRNAs, lncRNAs, and circRNAs in intestinal tissues from neonates with NEC. Compared to controls, they identified 281 differentially expressed mRNAs, 21 miRNAs, 253 lncRNAs, and 207 circRNAs in infants with NEC. Through RNA regulatory network construction, the pathogenic gene hexokinase 2 (HK2) was pinpointed. This hypoxia-associated gene may mediate carbohydrate metabolism dysregulation in NEC pathogenesis (38). Compared to non-NEC infants, neonates with NEC exhibit distinct plasma miRNA profiles. Specifically, miR-1290, miR-1246, and miR-375 serve as specific biomarkers of NEC, with miR-1290 demonstrating the highest diagnostic value for discriminating between medical and surgical NEC (39). In intestinal tissues of NEC, significant downregulation of miR-429/200a/b and miR-141/200c clusters was observed (40). These miRNA clusters demonstrated binding capacity to multiple target genes with concomitant gene expression modulation, exerting negative regulatory effects on VEGF, E-selectin (SELE), kinase insert domain receptor (KDR), fms-related tyrosine kinase 1 (FLT1), and hepatocyte growth factor (HGF) (40). Functional studies confirmed their involvement in three critical NEC associated pathways: inflammatory response (41), angiogenesis (42), and epithelial barrier dysfunction (43).

### 5.2 Mechanistic involvement of non-coding RNAs in NEC

Non-coding RNAs play regulatory roles in intestinal inflammation, apoptosis, damage, and repair processes. Among these, miRNAs have garnered the most research attention in NEC. miR-141-3p targets motor neuron and pancreas homeobox 1 (MNX1), alleviating intestinal inflammation and oxidative stress in NEC murine models (44). Mechanistically, it protects IECs from LPS-induced injury by suppressing RIPK1-mediated inflammatory responses and necroptosis (45). miR-124 promotes IECs apoptosis and inflammatory cell infiltration by targeting ROCK1 to inhibit the expression of TLR9 (46). Human milk contains diverse non-coding RNAs, and the protective role of breast milk-derived non-coding RNAs against NEC has been demonstrated. Studies reveal that exosomal miRNAs from breast milk suppress the TLR4/NF-κB signaling pathway, mitigating intestinal inflammation and oxidative stress to reduce NEC risk (47). Notably, preterm breast milk exosomes exhibit superior capacity to enhance intestinal epithelial repair compared to term breast milk exosomes. This functional advantage coincides with the enrichment of lncRNAs in in preterm milk exosomes (48). Bioinformatic analyses suggest that these enriched lncRNAs may modulate proliferative signaling pathways, including JAK-STAT and AMPK (48). Collectively, these findings suggest that miRNAs may serve as key mechanistic regulators of NEC pathogenesis, while also discovering the therapeutic potential of milk-derived non-coding RNAs.

## 6 Crosstalk of epigenetic mechanisms in NEC

*In vitro* studies reveal that LPS-hyporesponsive intestinal epithelial cell lines exhibit significantly higher histone deacetylation and DNA methylation at the TLR4 locus compared to monocytic cell lines or LPS-hyperresponsive epithelial cells, suggesting that TLR4 responsiveness to LPS is modulated by DNA methylation and histone acetylation (27). LPS stimulation induces dynamic epigenetic remodeling at the COX-2 promoter in intestinal epithelial cells, characterized by sequential DNA hypermethylation, histone acetylation, and histone methylation. Specifically, LPS promotes COX-2 DNA methylation, followed by JMJD3 recruitment to the promoter region, which mediates H3K27 demethylation and ultimately upregulates COX-2 expression (49). Both TLR4 and COX-2 are overexpressed in NEC and contribute to its pathogenesis (40). Furthermore, VEGF dysregulation in NEC involves dual epigenetic mechanisms: aberrant DNA methylation at its promoter and and post-transcriptional repression by specific miRNAs (e.g., miR-429/200a/b and miR-141/200c) (19, 40). This combined regulation leads to VEGF downregulation in NEC (50), while exogenous VEGF supplementation attenuates NEC progression (51). Breast milk-derived exosomal miR-148a-3p exerts protective effects in NEC by binding the 3'UTR of the tumor suppressor TP53, suppressing p53 expression and upregulating the deacetylase SIRT1 via the p53/SIRT1 axis (52). Additionally, Gao et al. (53) identified the lncRNA LINC01512 as a novel regulator of SIRT1, which enhances Treg immunosuppressive function while inhibiting Th17 pro-inflammatory activity. NEC tissues exhibit significant LINC01512 downregulation, inversely correlating with Treg/Th17 imbalance (53). SIRT1 further mitigates inflammation by inhibiting NF-κB acetylation and nuclear translocation (54). Therefore, the miR-148a-3p/p53/SIRT1 and LINC01512/SIRT1 axes represent promising anti-inflammatory targets for NEC.

## 7 RNA modification

**Figure 2:**
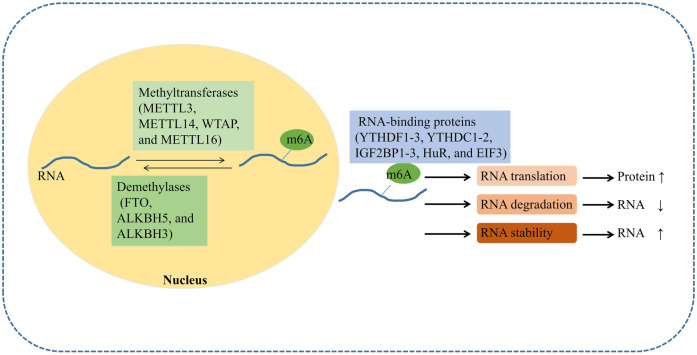
Mechanism of m6A modification. N6-methyladenosine (m6A) modification is dynamically regulated by three classes of proteins: writers (methyltransferases), erasers (demethylases), and readers (m6A-binding proteins). Readers directly mediate downstream effects, including RNA translation, degradation, and stability by recognizing m6A.

In addition to the above classic epigenetic mechanisms, N6-methyladenosine (m6A), the most abundant RNA modification in mammals, has emerged as a key regulator in gut (patho) physiology (55). The modifications predominantly occur near the stop codon region and the 3′-untranslated region (3′-UTR) of RNA, regulated by three classes of enzymes: methyltransferases (e.g., METTL3, METTL14, WTAP, and METTL16), demethylases (e.g., FTO, ALKBH5, and ALKBH3), and RNA-binding proteins that regulate RNA translation, degradation, and stability (e.g., YTHDF1-3, YTHDC1-2, IGF2BP1-3, HuR, and EIF3) (55) (Figure 2). The m6A modification regulates mRNA abundance, spatiotemporal dynamics, and alternative splicing processes, thereby orchestrating protein expression profiles and biological phenotypes. m6A modification exerts essential regulatory functions in fundamental cellular processes including development, tissue regeneration, immune homeostasis, and damage response mechanisms (56).

### 7.1 Characteristics of m6A modification in clinical intestinal diseases

**Figure 3:**
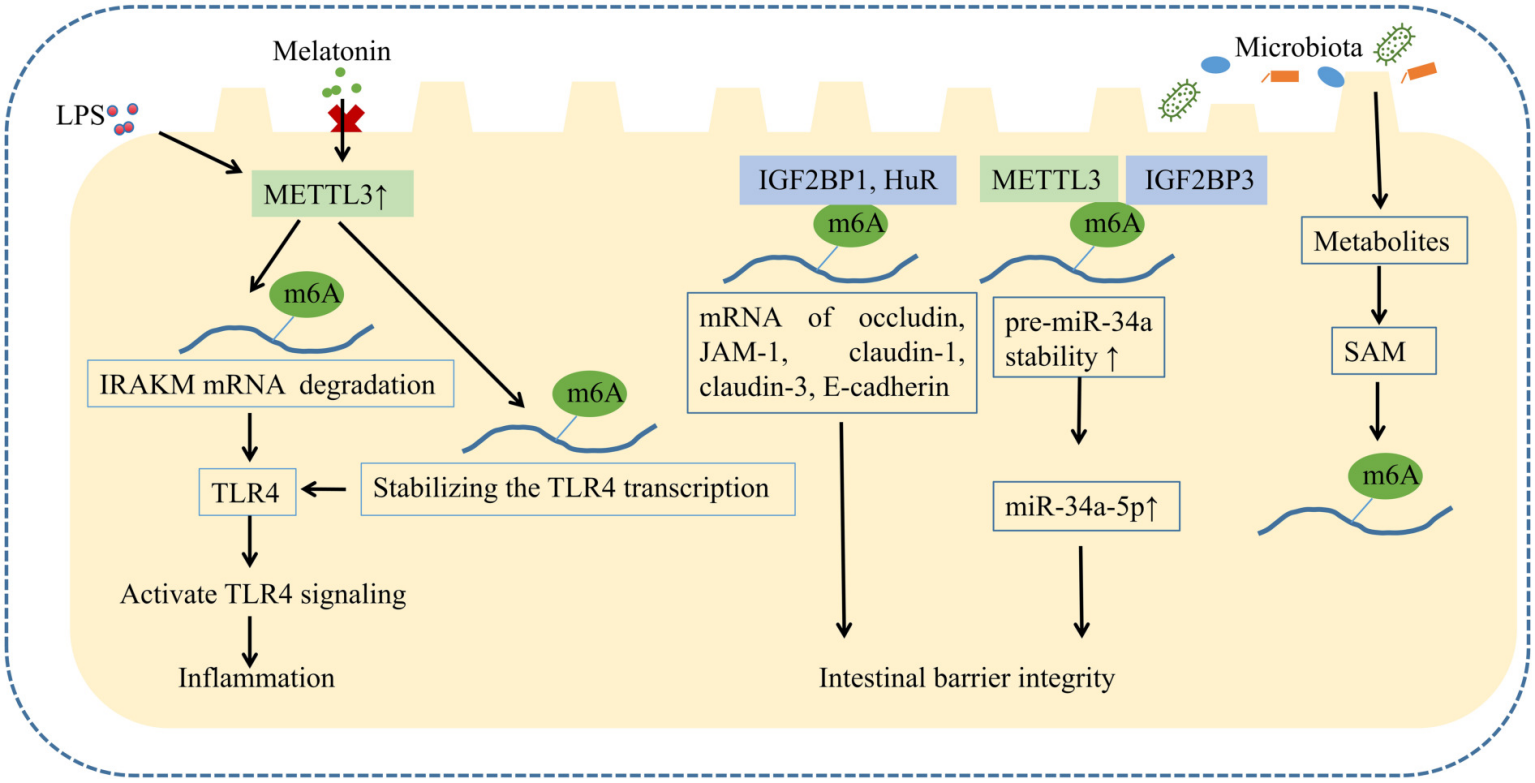
m6A modification in intestinal epithelial cells. Abundant m6A modifications exist in intestinal epithelial cells. These modifications are regulated by the gut microbiota and its metabolites, and they play roles in intestinal inflammation and barrier function, either individually or in combination with other epigenetic mechanisms such as miRNA.

Clinical studies have demonstrated disease-specific m6A epitranscriptomic signatures across intestinal pathologies, where dynamic dysregulation of m6A regulators reflects disease progression stages. Inflammatory bowel disease (IBD) exhibits paradoxical m6A regulatory dynamics: METTL3 and FTO demonstrate elevated expression concomitant with METTL14 and HuR downregulation (57). Notably, conflicting evidence delineates activity-dependent fluctuations of these regulators, characterized by the upregulation of METTL3 and FTO during the active phases, in contrast to their downregulation during the remission (56). This coordinated dysregulation of methyltransferases and demethylases underscores the intricate crosstalk within m6A regulatory networks (57). As principal IBD subtypes, ulcerative colitis (UC) and Crohn's disease (CD) demonstrate clinical heterogeneity in m6A regulator expression. UC patients exhibit serum METTL3 and WTAP levels correlating with disease extent, subtype and IL-6, whereas CD patients show METTL3 associations with lesion location and Crohn's Disease activity index, but no significant WTAP variation vs. controls (58). The partial mechanism of m6A modification in intestinal injury is summarized in Figure 3.

### 7.2 m6Amodification and intestinal inflammation

The pivotal role of m6A modification in intestinal inflammation has been corroborated across *in vivo* and *in vitro* experimental models. Mechanistically, methyltransferases METTL3 and METTL14 exhibit upregulated expression in LPS induecd IECs (59). Notably, METTL3 exacerbates LPS-induced inflammatory cascades by potentiating epithelial apoptosis, elevating oxidative stress burden, and impairing antioxidant defenses (60). *In vitro*, METTL3 promotes TLR4 signaling by enhancing m6A modification of IRAKM mRNA, a negative regulator of TLR4, thereby accelerating its degradation (61). METTL3-deficient macrophages exhibit reduced TNF-α production upon LPS stimulation *in vitro*, indicating that METTL3-mediated m6A modifications activate macrophage responses (61). In LPS induced endotoxemia, METTL3 directly modulates m6A on TLR4 mRNA, promoting its translation and stabilizing the transcript, which elevates TLR4 protein expression and activates the neutrophil TLR4 signaling pathway. Ultimately, CXCR2 activation and internalization, dependent on the TLR4 signaling pathway, drive neutrophil mobilization from the bone marrow into systemic circulation (62). Mice with intestinal epithelial METTL3 deficiency resist rotavirus infection via m6A-dependent stabilization of Irf7 mRNA, which enhances type I and III interferon production (63). Olazagoitia-Garmendia et al. (64) identified elevated m6A levels in the 5′-UTR of XPO1 mRNA in celiac disease patients, where hypermethylation increases XPO1 protein expression *in vitro* and *in vivo*, driving NF-κB activation and inflammation. Cumulatively, these findings elucidate the pivotal role of METTL3 in exacerbating inflammatory responses through m6A-mediated epitranscriptomic regulation, particularly via modulation of the TLR4/NF-κB signaling axis (64). Li et al. (65) confirmed that increased m6A methylation correlates with colitis development *in vivo*. Whereas *in vitro*, melatonin attenuates LPS-induced colonic inflammation by suppressing METTL3 and reducing m6A levels via melatonin receptor 1B (MTNR1B) *in vitro*. Despite growing evidence linking m6A regulators to inflammation, conflicting studies underscore the complexity of m6A regulation. Cai et al. (66) reported reduced global m6A levels and METTL3 expression in LPS-treated macrophages. METTL3 downregulation significantly upregulated pro-inflammatory cytokines (e.g., TNF-α, IL-6, NO), potentially by stabilizing NOD1 and RIPK2 mRNAs, amplifying NOD1 signaling, and exacerbating inflammation.

### 7.3 m6A modification and intestinal cell survival

m6A modification plays a critical role in balancing intestinal cell renewal, differentiation (67), death, and survival (25, 68). Huang et al. (69) observed apoptotic and necrotic markers, including cleaved caspase-3 (c-caspase 3) and phosphorylated MLKL (p-MLKL), in METTL3 knockout IECs. Both necroptotic and apoptotic cells were enriched in the crypt region, potentially linked to METTL3 enrichment in crypts (70). *In vivo*, the abundant necroptotic and apoptotic cells in stem and transient amplifying cells but not in Paneth cells (69). Treg cells lacking METTL3 in intestinal epithelium exhibited loss of m6A modification on SOCS mRNA, leading to increased SOCS mRNA stability and expression, which suppressed the IL-2-STAT5 signaling pathway and impaired Treg-mediated anti-inflammatory capacity. Consequently, METTL3-deficient Treg mice developed severe autoimmune diseases (71). METTL14-mediated m6A modification of GsdmC mRNA is essential for the viability of Lgr5+ colonic stem cells, ensuring proper mucosal development (72). METTL14 deletion induced colonic stem cell apoptosis, causing mucosal barrier dysfunction and severe colitis, partly by regulating Nfkbia mRNA stability and the NF-κB pathway (68). Ito-Kureha et al. (73) reported that WTAP-deficient T cells in mice developed colitis at 7 weeks of age, accompanied by upregulated RIPK1 expression and apoptosis. Conversely, WTAP knockdown suppressed M1 macrophage polarization and CD4+ T cell infiltration, alleviating UC progression in mice (74). These findings highlight cell type-specific roles of m6A modification, sometimes exerting opposing effects.

### 7.4 m6A modification and intestinal barrier

Emerging evidence indicates that m6A regulates intestinal barrier integrity, encompassing mechanical, chemical, and immune barriers. Li et al. (75) demonstrated that METTL3 knockdown reduced ZO-1, occludin, and claudin-3 expression in IECs, compromising barrier function. METTL3 also modulates β-defensin production after E. coli infection by enhancing GPR161 transcription via m6A modification, thereby promoting defensin expression to counteract bacterial invasion (76). m6A readers IGF2BP1 and HuR directly bind to tight junction protein mRNAs (e.g., occludin, JAM-1, claudin-1, claudin-3, E-cadherin), enhancing their stability and translation to reinforce barrier integrity. IGF2BP1 deficiency exacerbated DSS-induced colitis by impairing epithelial barrier function (77). Intestinal HuR knockout mice exhibited reduced Paneth cell numbers and lysozyme granules, with HuR regulating Paneth cell function via TLR2 membrane localization (78). METTL14 deficiency diminished goblet cell-derived mucins (MUC2, TFF3), exacerbating tight junction dysfunction (72). Li et al. (75) demonstrated that mesenchymal stem cells improve intestinal barrier function in ischemia-reperfusion injury via exosomal miR-34a-5p secretion. Mechanistically, METTL3/IGF2BP3 stabilizes pre-miR-34a through m6A modification, thereby enhancing mature miR-34a-5p expression (75). NEC is characterized by compromised intestinal barrier integrity, impaired mucosal repair (9), Paneth cell depletion, and dysfunctional Paneth cell activity (79), suggesting that m6A-mediated regulation of intestinal barrier homeostasis may contribute to NEC pathogenesis.

### 7.5 m6A modification and gut microbiota

m6A modification exhibit bidirectional crosstalk with gut microbiota. Microbial communities modulate host mRNA m6A patterns, as evidenced by germ-free mice showing elevated m6A levels in intestinal, hepatic, and cerebral tissues compared to conventional counterparts (80). Microbial metabolites including folate, betaine, choline, vitamin B12, fumarate, succinate, and butyrate remodel one-carbon metabolism, methionine cycling, and the tricarboxylic acid cycle to regulate SAM and α-ketoglutarate production, critical substrates for methylation and demethylation processes, thereby dynamically shaping m6A landscape (81). During early postnatal development, gut microbiota maturation (e.g., folate-producing *Lactobacillus* and *Bifidobacterium*) enhances intestinal DNA methylation and RNA m6A modifications, ensuring proper gut maturation (80, 82). During the critical postnatal developmental window, the gastrointestinal microbiota undergoes dynamic maturation characterized by expanding taxonomic diversity and functional abundance, establishing spatiotemporal microbiota-host crosstalk (83). Conversely, host m6A modification can modulate microbial homeostasis. METTL14 knockout mice maintain baseline microbiota composition at 4 weeks but develop significant dysbiosis by 24 weeks, characterized by depletion of *S24-7* and *Lachnospiraceae* with concurrent expansion of *Bacteroidaceae*, *Spirochaetaceae*, *Deferribacteraceae*, *and Enterobacteriaceae* (84). *In vivo*, FTO deficiency restructures gut microbiota composition by enhancing Lactobacillus abundance and suppressing *Porphyromonadaceae* and *Helicobacter*, thereby attenuating intestinal inflammation (85). FTO critically governs protein-energy homeostasis, with essential amino acids (EAAs) serving as potent stimulators of its expression. Notably, formula milk contains threefold higher branched-chain EAAs (BCAAs) compared to breast milk, potentially hyperactivating FTO signaling (86). Therefore, the overexpression of FTO may be one of the mechanisms by which formula feeding increases the risk of NEC.

## 8 Conclusion

In conclusion, accumulating evidence validates the involvement of multiple epigenetic mechanisms in NEC pathogenesis. Notably, the pivotal role of m6A modification in intestinal homeostasis underscores its potential to drive NEC development. Epigenetic modifications orchestrate gene expression across transcriptional and post-transcriptional tiers, modulating NEC susceptibility and progression. These findings highlight promising therapeutic targets for NEC intervention.
