# Correction: Epigenetics in neonatal necrotizing enterocolitis: current understanding and the potential involvement of m6A modification

**DOI:** 10.3389/fped.2025.1704225

**Published:** 2025-10-08

**Authors:** Yixian Chen, Yujun Chen

**Affiliations:** ^1^ Neonatology, Liuzhou Hospital of Guangzhou Women and Children’s Medical Center, Guangxi, China; ^2^ Department of Pediatrics, The Second Affiliated Hospital of Guangxi Medical University, Guangxi, China

**Keywords:** necrotizing enterocolitis, epigenetics, N6-methyladenosine, neonates, DNA methylation

Author **Yujun Chen** was erroneously assigned to **Affiliation 1**. This affiliation has now been removed for the author. **Affiliation 2** was omitted for author **Yujun Chen**. This affiliation has now been added for the author.

The original version of this article has been updated.

